# Supplementary material for: Additions to the phylogeny of colubrine snakes in Southwestern Asia, with description of a new genus and species (Serpentes: Colubridae: Colubrinae)
Source: PeerJ. 2020 Apr 21;8:e9016. doi: 10.7717/peerj.9016 (PMC7182026; doi:10.7717/peerj.9016)
Supplement: Table S1 [file peerj-08-9016-s001.docx]

| **Species** | **Locality** | **Voucher** | **Gene** | **GenBank A.N.** |
| --- | --- | --- | --- | --- |
| *Hierophis andreanus* (reassigned to *Dolichophis* *andreanus*) | Iran | ICSTZM.7H.1154 | ND4 | MN531564 |
| *Hierophis andreanus* (reassigned to *Dolichophis* *andreanus*) | Iran | ICSTZM.7H.1154 | CYTB | MN531565 |
| *Hierophis andreanus* (reassigned to *Dolichophis* *andreanus*) | Iran | ICSTZM.7H.1154 | 12S | MN536808 |
| *Persiophis fahimii* **Gen. et sp. nov.** | Iran | ICSTZM.7H.1151 | ND4 | MN531566 |
| *Persiophis fahimii* **Gen. et sp. nov.** | Iran | ICSTZM.7H.1151 | CYTB | MN531567 |
| *Persiophis fahimii* **Gen. et sp. nov.** | Iran | ICSTZM.7H.1151 | 12S | MN536809 |
| *Persiophis fahimii* **Gen. et sp. nov.** | Iran | ICSTZM.7H.1151 | RAG2 | MT163746 |
| *Persiophis fahimii* **Gen. et sp. nov.** | Iran | ICSTZM.7H.1151 | NT3 | MT163747 |
| *Persiophis fahimii* **Gen. et sp. nov.** | Iran | ICSTZM.7H.1151 | CMOS | MT163748 |
| *Persiophis fahimii* **Gen. et sp. nov.** | Iran | ICSTZM.7H.1151 | BDNF | MT163749 |
