# Supplementary material for: Additions to the phylogeny of colubrine snakes in Southwestern Asia, with description of a new genus and species (Serpentes: Colubridae: Colubrinae)
Source: PeerJ. 2020 Apr 21;8:e9016. doi: 10.7717/peerj.9016 (PMC7182026; doi:10.7717/peerj.9016)
Supplement: Table S2 [file peerj-08-9016-s002.docx]

| Species | Number of Projections | Voxel Size (µm) |
| --- | --- | --- |
| *Hierophis andreanus* (reassigned to *Dolichophis* *andreanus*) | 840 | 18.356 |
| *Persiophis fahimii* **gen. et sp. nov.** | 841 | 19.212 |
