# Supplementary material for: Additions to the phylogeny of colubrine snakes in Southwestern Asia, with description of a new genus and species (Serpentes: Colubridae: Colubrinae)
Source: PeerJ. 2020 Apr 21;8:e9016. doi: 10.7717/peerj.9016 (PMC7182026; doi:10.7717/peerj.9016)
Supplement: Table S3 — Abbreviations: Pt, Pterygoid teeth (+ = present, 0 = absent); Pr, Premaxilla (N = Normal, P = projected); Mx, Large diastema on the middle maxilla (+ = present, 0 = absent) [ops = opisthoglyphous]; T, Tooth shape (N = Normal, V = small and vestigial); 1L, First supra labial scale (≤ = smaller or eqqual to the second supra labial, > bigger than the second supra labial); R, Rostral scale (N = Normal, P = Projected); Dor; Number of dorsal scales at midbody; Pupil (R = round, V = vertical, H = horizontal). Data are based on (Boulenger, 1898); (Bourgeois, 1968); (Broadley, 1994); Broeckhoven & du Plessis, 2017; (Chan-Ard, Nabhitabhata & Parr, 2015); (Chippaux & Jackson, 2019); (Das et al., 2019); (Gans, 1954); (Kardong, 1979); (Kharin & Akulenko, 2008); (Mahlow et al., 2013); (Marx, 1959); (Mirza & Patel, 2018); (Mirza et al., 2016); (Nguyen et al., 2020); (Poyarkov, Nguyen & Vogel, 2019); (Rajabizadeh, 2018); (Saleh & Sarhan, 2016); Schätti, 1985, 1987; (Tsai & Mao, 2017); (Utiger, Schätti & Helfenberger, 2005); (Wade, 2008); Wagner & Böhme, 2007; (Wallach, Lanza & Nistri, 2010); (Wang, Shi & Guo, 2019). [file peerj-08-9016-s003.docx]

| **Genus** | **Pt** | **Pr** | **Mx** | **T** | **1SL** | **R** | **Dor** | **P** |
| --- | --- | --- | --- | --- | --- | --- | --- | --- |
| ***Aeluroglena*** | ? | N | 0 | N | ? | N | 21 | V |
| ***Aprosdoketophis*** | + | N | 0 | N | ≤ | N | 19 | R |
| ***Archelaphe*** | ? | ? | ? | ? | < | N | 19 | R |
| ***Argyrogena*** | + | N | 0 | N | < | N | 21-23 | R |
| ***Boiga*** | + | N | 0+ | N | < | N | 19-27 | V |
| ***Coelognathus*** | + | N | 0 | N | ≤> | N | 21-29 | R |
| ***Coronella*** | + | N | 0 | N | < | N | 19-21 | R |
| ***Crotaphopeltis*** | + | N | 0 | N | ≤ | N | 17-21 | V |
| ***Dasypeltis*** | 0 | N | + | V | < | N | 18-28 | V |
| ***Dipsadoboa*** | ? | ? | ? | N | < | N | 17-19 | V |
| ***Dispholidus*** | + | N | 0 | V | < | N | 19-21 | R |
| ***Dolichophis*** | + | ≈P | 0 | N | < | N | 17-19 | R |
| ***Eirenis*** | + | N | 0 | N | < | N | 15-19 | R |
| ***Elachistodon*** | +(v) | N | + | VN | < | N | 15 | R |
| ***Elaphe*** | + | N | 0 | N | ≤> | N | 19-29 | R |
| ***Euprepiophis*** | ? | ? | ? | N | < | N | ?21-23 | R |
| ***Gonyosoma*** | + | ? | 0 | N | ≤> | NP | 19-27 | R |
| ***Hapsidophrys*** | ? | ? | 0 | N | ≤ | N | 15 | R |
| ***Hemorrhois*** | + | N | 0 | N | < | N | 21-25 | R |
| ***Hierophis*** | + | ≈P | 0 | N | < | N | 19 | R |
| ***Liopeltis*** | + | N | 0 | N | ≤ | N | 13-17 | R |
| ***Lycodon*** | + | N | 0+ | N | ≤ | N | 17 | R |
| ***Lytorhynchus*** | +,0 | P | 0+ | N | < | P | 19-21 | R |
| ***Macroprotodon*** | + | N | + | N | < | N | 19-25 | R,Oval |
| ***Meizodon*** | + | ? | 0 | N | ≤ | N | 19-21 | R |
| ***Muhtarophis*** | + | P | 0 | NV | < | P | 17 | R |
| ***Oligodon*** | + | P | 0(ops) | N | < | P | 13-21 | R |
| ***Oreocryptophis*** | + | N | 0 | N | < | N | 19 | R |
| ***Orientocoluber*** | + | N | 0 | N | < | N | 19 | R |
| ***Persiophis* gen. nov.** | 0 | N | 0 | V | > | N | 15 | R |
| ***Philothamnus*** | ? | ? | 0(ops) | N | < | N | 13-15 | R |
| ***Platyceps*** | + | N | 0 | N | < | N | 19 | R |
| ***Ptyas*** | + | N | 0 | N | < | N | 16-17 | R |
| ***Rhynchocalamus*** | 0 | Rev | 0 | N | < | P | 15 | R |
| ***Scaphiophis*** | + | P | 0 | N | < | P | 19-31 | R |
| ***Spalerosophis*** | + | N | 0 | N | < | N | 25-43 | R |
| ***Telescopus*** | + | N | 0 | N | < | N | 17-23 | V |
| ***Thelotornis*** | ? | ? | 0(ops) | N | > | P | 19 | H |
| ***Thrasops*** | ? | N | 0(ops) | ? | < | N | 13-21 | R |
| ***Toxicodryas*** | + | ? | 0 | N | < | N | 19-25 | R |
| ***Wallaceophis*** | + | N | 0 | N | < | N | 23 | R |
| ***Zamenis*** | + | N | 0 | N | < | N | 19-29 | R |
| ***Wallophis*** | + | N | 0 | N | < | N | 23 | R |
