# Supplementary figures and images for: Additions to the phylogeny of colubrine snakes in Southwestern Asia, with description of a new genus and species (Serpentes: Colubridae: Colubrinae)
Source: PeerJ. 2020 Apr 21;8:e9016. doi: 10.7717/peerj.9016 (PMC7182026; doi:10.7717/peerj.9016)

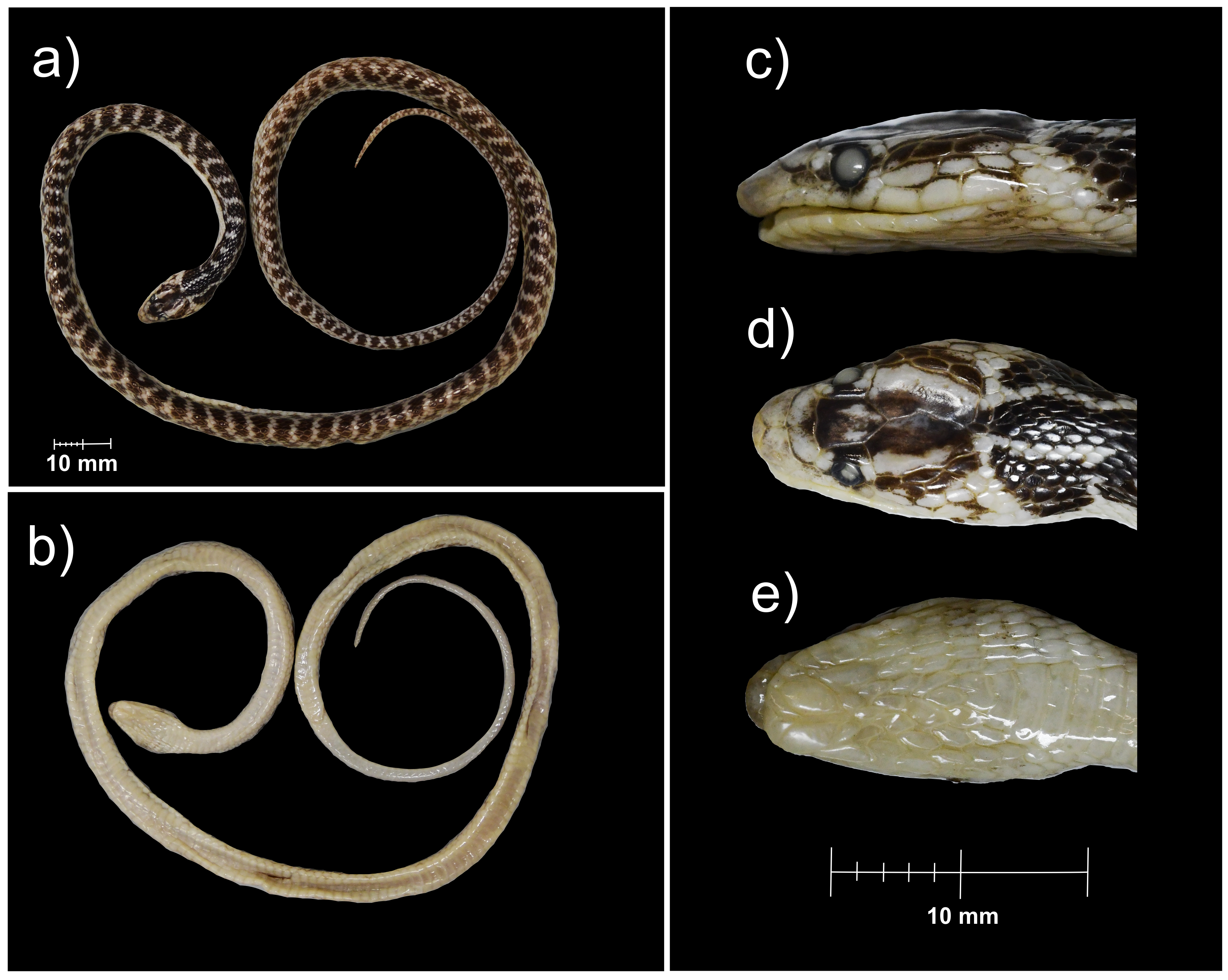

Supplement: Figure S1 — (a) Dorsal and (b) ventral views of the whole body; details of head scalation in close-up (c) dorsal, (d) lateral and (e) ventral views. Photos by Dominique Adriaens. [file peerj-08-9016-s004.png]
