# Supplementary material for: Additions to the phylogeny of colubrine snakes in Southwestern Asia, with description of a new genus and species (Serpentes: Colubridae: Colubrinae)
Source: PeerJ. 2020 Apr 21;8:e9016. doi: 10.7717/peerj.9016 (PMC7182026; doi:10.7717/peerj.9016)
Supplement: Data S1 [file peerj-08-9016-s005.pdf]

NEWLY OBTAINED mtDNA SEQUENCES OF COLUBRID SNAKES

>MN531564_Hierophis_andreanus_ICSTZM.7H.1154_ND4

ATCCAAACACAATGAAGCCTCTCAGGCGCCATAGCCCTAATAATCGCCCACGGATTCACTTCTTCAGCACTTTTTTG
TCTAGCCAATACCACCTATGAACGAACCAAAACTCGAATTATGATTCTCACACGAGGATTTCACAACATCTTACCAA
TACTAACAACCTGGTGACTACTAACTAACCTTATAAATATTGCAACCCCACCAAGCATAAATTTTACAGGAGAACTA
CTAATCGTATCATCACTATTCAACTGATGTCCCACAACAATCATTATATTCGGACTGTCAATACTTATCACAGCATCA
TACTCACTGCACATATTCCTATCAACACAAATAAACACACCCGCATTAAGTACTACAACACAACCAACACATTCACG
CGAACATCTGACTATAACACTCCACATTATCCCACTAATCCTAATCTCATTAAAACCAGAGCTAGTTATT

>MN531565_Hierophis_andreanus_ICSTZM.7H.1154_CYTB

CACACACTAATACTATTCAACCTCCTACCAGTAGGATGCAACATCTCAACCTGATGAAACTTCGGATCCATGCTATT
AACCTGCTCAACCCTACAAATCCTTACTGGCTTCTTTCTAGCTATTCACTATACAGCCAACATCAACTTACCCTTCTCA
TCTATCGTACATATTACACGAGACGTACCCTACGGATGAATAATACAAAACCTTCATGCAATTGGCGCATCTATATT
CTTCATCTGTATCTACATCCACATTGCTCGAGGACTTTACTACGGATCTTACCTAAATAAAAATGTGTGATTATCAG
GAGTTACCCTAATAATTATTCTCATAGCAACAGCCTTCTTCGGATATGTATTACCCTGAGGACAAATATCATTCTGA
GCAGCAACAGTAATTACTAATCTACTAACAGCTATCCCATATATTGGCACAACACTAACTACATGGTTGTGAGGCG
GATTCTCAATCAATGACCCGACCCTAACCCGATTCTTTGCCCTACACTTCATCCTACCATTTACCATTATCTCCATATC
TTCAATCCATATCATACTCCTACATACAGAGGGATCTAGTAACCCACTAGGAACAAACTCAGATATCGACAAAATCC
CATTTCACCCCTACCACTCCTATAAAGACGCTATAATACTTACCACCATAATAACAGCACTGTTCCTAATCATATCAT
TCACCCCAGATATCTTCAATGACCCAGAAAACTTCTCAAAAGCCAACCCCCTAGTAACACCACAACACATCAAACCA
GAATGATACTTCCTATTTGCCTATGGAATCCTACGATCAATTCCAAACAAATTGGGCGGAACAATAGCTCTTATACT
CTCCGTAGCAATTCTACTAACAGCACCCTATACCCACACCTCACACCTCCGCCCCATAACATTTCGCCCACTAATACA
ACTAAAATTCTGAACCCTAGTTGCTACATTTATCACAATTACATGAACAGCCACTAAACCAGTAGAACCACCATTT

>MN536808_Hierophis_andreanus_ICSTZM.7H.1154_12S

ATTAATCCAACCCCCCCTAGCCTAACAGTCTATATACCGCCGTCGCCAGCATACCTTCTGAAAGAAATAAAGTAAGC
TAAACAGTTACCACACTAACACGACAGGTCGAGGTGTAACTAATGGGCGGGTCAAGATGGGCTACATTTTCTAAA
ATAGAAAATACGAACTAAACTATGAAAAAGAAACTGAAGGCGGATTTAGCAGTAAATTAAGGACATAATACTTAA
TCGAAATTAATGCAATGAGGTGCGTACACACCGCCCGTC

>MN531566_Persiophis_fahimi_ICSTZM.7H.1151_ND4

TGAAGCCTATCAGGCGCCATAGCCCTAATAATCGCCCACGGATTCACCTCTTCAGCACTTTTCTGCCTAGCCAACAC
CACCTATGAACGAACTAATACTCGTATCATAATTCTTACACGAGGATTCCACAATATTTTACCAATACTAACAACCT
GATGACTACTAACCAACCTAATAAACATTGCAACACCACCCAGCATAAACTTCACCGGGGAACTATTAATCGCCTC
ATCACTATACAACTGATGCCCAGCAACAATCATCATATTCGGACTCTCAATACTTATCACAGCATCCTACTCTCTACA
TATACTCTTATCAACACAAACTGGAACACCACTACTAAACTCAATAACACACCCAACACACTCACGAGAACATCTCC
TAATAACGCTCCACATTATACCACTAATACTTATCTCTCTCAAACCAGAACTAATCTTT

>MN531567_Persiophis_fahimi_ICSTZM.7H.1151_CYTB

ATACTCATACTATTTAACCTACTCCCAGTAGGATCAAACATTTCAACATGATGAAATTTCGGATCCATACTACTAACC
TGCTCTGCCCTACAAATTATGACCGGATTCTTCCTAGCCATCCACTATACAGCCAACATCAATCTCGCCTTCTCATCT
GTTATCCACATCACACGAGATGTGCCATATGGATGAACTATACAAAATCTACATGCAATCGGCGCATCCATATTTTT
CATCTGCATCTATATCCACATCGCACGTGGACTATACTACGGATCCTACCTTAATAAAAATGTGTGACTATCAGGAA
CTATCCTACTAATTATCCTAATAGCAACAGCCTTCTTCGGCTACGTACTACCATGAGGACAAATATCATTCTGAGCC

GCAACAGTAATCACTAACCTACTAACAGCCCTCCCATATGTTGGCACAATACTAACCACCTGATTATGGGGGGTT
TCTCAATTAATGACCCCACTCTAACCCGATTCTTTGCCCTCCACTTCATCTTACCATTCACCATTATCTCAATATCTTC
AATCCATATTATACTTCTACACACAGAAGGCTCCAGCAACCCCCTAGGAACAAACTCAGACATTGATAAAATCCCAT
TTCACCCATACCACTCCCATAAAGATCTCCTTATACTAACCATCATAATCACTGCACTATTCACCATTATATCATTTAC
CCCAGACATATTCAATGACCCAGAAAACTTCTCAAAAGCCAACCCCCTGGTAACACCACAACACATCAAACCAGAG
TGATATTTCTTGTTTGCCTACGGAATCCTTCGATCTATCCCAAATAAGCTGGGAGGAACCATAGCCCTAGTTATATC
CGTAATAATCCTAATAACAATACCATTCACACACACCTCACACATACGACCAATAACTTTCCGCCCCCTAGCACAAC
TAAAATTTTGAACCTTAGTAGCTACATTCATCACAATTACGTGAGCAGCCACCAAACCAGTAGAACCTCCATTTAC

>MN536809_Persiophis_fahimi_ICSTZM.7H.1151_12S

TAGATACCCCACTATGCCTAACTGTAACCCAACAATTAAATTACCAATTGTTCGCCAAATAACTACGAGTAAAAACT
TAAAATTTAAAAGACTTGACGGTACTTCACAACACCCTAGAGGAGCCTGTCCAGTAACCGATACTCCACGATCAAC
CCAACCCTTTCTAGCCCAACAGTCTATATACCGCCGTCGCCAGCCTACCTTATGAAAGAAACAAAGTAAGCCAAAT
AGTCACACACTAACACGACAGGTCGAGGTGTAACCAATGAAGGGGGCAAAGATGGGCTACACTCTCTAACGCAG
AGAACACGAATAAACTTTGAAATAAGAAACTGAAGGTGGATTTAGCAGTACACTAAGAACAAACATACTTGGTCG
AAATTAATGCAATGAAGTGCGTACACACCGCCCGTC

NEWLY OBTAINED nuDNA SEQUENCES OF *Persiophis fahimi*

>MT163746_Persiophis_fahimi_ICSTZM.7H.1151_RAG2

GTTAATGTGGTTCACAGTCTAGGCAAAAGTATGATAGCTATAATTGGGGGACGATCTTACATAGCTCTTGGACAGA
GGACAACTGAAAAATGGAATAGTGTGGTTGACTGTATGCCACATATATTTCTGGTTGACCCTGAATTCGGATGTTG
TACTTCATATGCTCTCCCAGAATTTCAGAATGGCTTTTCTTTTCATCTCTCTCTTGTCCAAAATGATATCATCTATATC
ATAGGAGGACATTCCCTGGAAAATAATATCAGACCTCCCAATTTCTATAAAATAAAAATTGATCTTCCCTTAGGCAG
TCCAGCTGTGAGCTGTGTAATTTTGCCTGGAGGAATCTCGGTCTCCAGTGCCATCATGACACAGACCAGAGAAAA
GGAATTTGTTGTTGTAGGAGGTTACCATTCTGACAACCAGAAAAGGATGATCTGTAATACTATCAACCTAGATGAT
AACAAAATAGAGATAGTGGAAACAGAGACACCAGAATGGACTCCAGATATCAAGCACTGCAAGACATGGTTTGG
GAGTGATATGGGAAATGGATCCATTTTATTTGGTATACCAGGAGACAATAGGCAGCTGACTTCAGATGCAAACTA
TTTCTACATATTAAAATGTCAAGTAGAAAATGGTCAGAGCTTAGAACAGATGGCACAAGTATGTAGTCAGAGCTCT
ACAGAAGATGCTGGAGAGTCCACTCCTTTT

>MT163747_Persiophis_fahimi_ICSTZM.7H.1151_NT3

CTGGCTTTTCTCTGTGGCATCCAATCGACCAGCATGGACCAAGGGAGTTTATCAGAAGATTCCATGAATTCTTTCCT
TAAAACATTGATTCAAGCTGGCATTTGGAAAAACAAGCAGACGGCCAGAACTAAAGACGGCGCGCCAACCACAGT
GAAGAAAACCGAGGTGGAACCAGAGTTGACTACAAGCCAAGATATTCGATTGGGTTTCCAGCCCGTTGTTTCGTT
GGATGCAGAATTACTGAGGCAGCAGAGACGTTTCAGTTCTCCACGGGTCCTCCTGAGTGAAAATACCCCCCTGGA
ACCCCCTCCTTTGTATCTGATGGAGGAGCCCATGGTGCTGAACCGAACGTCTCGCCGGAAGAGGTATACTGAAGG
GAAAACTCACCGCGGGGAATATTCCGTGTGCGACAGCGAGAGCCGATGGGTCACGGACAAAACGTCCGCTGTGG
ACATCCGAGGACACCAGGTGACTGTCCTGGGTGAAATCCGAATGGGCCCATCTCCGGTCAAACAATATTTTTACGA
AACGAGATGTAAGCAAGCCAAGCCTGCCAAGAGCGGTTGTCGTGGTATCGACGACAAACACTGGAATTCCCAGTG
CAAAACCTCACAAACATTTGTGCGCGCA

>MT163748_Persiophis_fahimii_ICSTZM.7H.1151_CMOS

GAAAAGTTCTTTGGGGTGGGAAGCGTGTCTAGGATTCGTCGGCTACCCCCCCACTTAGCTTGGTGTTCAATCGACT
GGGATCAGTTATGTCTCCTGCATCTCCTCGGCTCTGGTGGGTTTGGTTCTGTTTACAAGGCAACTTACCATGGAGCT
ACAGTGGCTGTAAAACAAGTGAAGAGATGTAGTAAAAACCATTTGGCATCACGGCAAAGCTTCTGGGCAGAACTA
AATGTAGCACGTCTTGACCATAACAATGTGGTACACATAGTAGCTGCTAGCACATGTACCCCTACTAGTCAGGATA
GTTTGGGTACCATAATTATGGAATATGCAGGTAACTGCACTCTACATCACATTATCTATGGGACTGGTTATTTAACA
GGAAATAATGATGGCCTTATATGTGACCATGGGTTTTTGAGTACAGCTCAGGCTGTCATTTACTCCTATGATATTGT
GGCAGGATTAATGTTTCTCCATTCTCAGTTAATTGTGCATCTGGATTTAAAACCTGCTAACATATTCATAACAGAAC
ATAATGTTTGTAAGATTGGAGACTTTGGATGCTCCCAAAAGCTAGAAGATAGCATATCTTCAGGACGACATCTTTG
TCATCAAGGGGGAACATACACACATCGTGCTCCTGAACTTCTTAAAGGTGAGAAAATCACACCCAAG

>MT163749_Persiophis_fahimii_ICSTZM.7H.1151_BDNF

AGTTGCATGAAAGCTGCCCCCATGAAGGAAGTTAGTATCAGAGGACAAGGCAGCTTGGCTTATCCTGGTCTTCGG
ACACAGGGAAATCTGGAGACCCTCAGTGGGCCCAATGATGCCACCAGAGGATTAACATCTTTGGCAGACACTTTT
GAACATGTCATTGAGGAGCTCCTGGATGAGCAGCAGGTCATTCAACCCAGCAAGGAAAATAAGGATGCAGACTT
GTACTCATCACGGGTGATGCTAAGCAGTCAAGTGCCTTTGGAGCCTCCTCTGCTTTTCCTGCTCGAGGAGTATAAA
AACTACTTGGATGCCGCAAACATGTCCATGAGGGTCCGGCGCCATTCTGACCCTGCTCGCCGTGGGGAGCTGAGT
GTGTGTGACAGTACTAGTGAGTGGGTGACAGCAGCTGAAAAAAAGACTGCAGTAGACATGTCCGGAGCAACAGT
TACAGTCCTGGAAAAAGTCCCAGTGCCCAAAGGCCAACTGAAGCAATATTTTTATGAGACCAAGTGCAGCACGAA
GGGTTATGCAAAAGAAGGCTGTAGGGGCATAGACAAGAGGTACTGGAATTCCCAGTGCCGAACTACTCAGTCTTA
CGTCCGCGCTCTCACCATGGATAACAAAAAGAGAGTTGGATGGCGCTTTATAAGAATAGACACTTCCTGT
